# Ionic Circuits Powered by Reverse Electrodialysis for an Ultimate Iontronic System

**DOI:** 10.1038/s41598-017-14390-0

**Published:** 2017-10-25

**Authors:** Seok Hee Han, Seung-Ryong Kwon, Seol Baek, Taek-Dong Chung

**Affiliations:** 1 Department of Chemistry, Seoul National University, Seoul, 08826 Korea; 2 Advanced Institutes of Convergence Technology, Suwon-Si, Gyeonggi-do 16229 Korea

## Abstract

Despite numerous reports on iontronic devices, there has been no whole circuit working in aqueous media including even power source. Herein, we introduce complete ionic circuits powered by reverse electrodialysis (RED) for the first time without employing any electronic components. The RED-driven polyelectrolyte diode successfully shows rectification behavior which is verified by monitoring dynamic ion distribution through fluorescence in real-time. We can also turn on and off the voltage applied to the circuit, and apply an arbitrary voltage by precisely manipulating the pressure imposed to an elastic connection tube filled with electrolyte. Furthermore, this new concept containing ionic power source advances to a more sophisticated ionic OR logic gate. The proposed system paves the way to develop not only passive iontronic devices (e.g. current ionic diode), but active ones requiring a source of energy, particularly such as a neuron-like information processor powered by fully ionic systems, and thereby aqueous computers.

## Introduction

Solid-state electronics succeeded in splendid achievements over decades in terms of cost, speed, size and other performances. Currently, electronic information processing devices such as computers are generally manufactured in the form of highly integrated chips based on semiconductors^1^. Apart from marvelous processing speed and efficiency of the electronic appliances, however, there are still limitations in their use to expand into biological information processing. In biological system, particularly the nervous system, ions and molecules are major signal transmitters, whereas electrons and holes are responsible for carrying informative signals in electronics, which are not compatible with the physiological environment^2^. Instead, ionic circuits composed of polyelectrolyte gels and electrolytes in aqueous solution have been considered as an alternative for the purpose of controlling ionic signals and thus processing information with non-linear characteristics. This concept, so-called ‘iontronics’, using ions for signal transmission is located somewhere between solid-state electronics and biological systems^3^. In the wake of the first polyelectrolyte system proposed by Bockris and his co-workers^4^, Shashoua utilized polyelectrolyte junction for mimicking potential spikes found in neuronal membranes^5^. These achievements were followed by a number of reports on iontronic microfluidic circuits and studies related to ionic diodes^6–10^, ionic transistors (e.g. bipolar-junction type^11–14^, field effect type^15^), ionic logic gates^8,16^, nanofluidic devices including diodes^17–20^ and transistors^21–23^.

Iontronic devices were suggested to work ultimately as biomimetic information processors in biological environment with biocompatible interfaces allowing even bilateral communication with living organs. But there are significant obstacles which include power source to drive their ionic circuits. As a fundamental biological signal processing unit, the neuron maintains the membrane potential by itself with its inherent chemical energy, which is unimaginable for current iontronic devices based on external electronic power sources. All the available electronic powers must involve electrodes that should have as low interfacial resistance as possible, e.g. Ag/AgCl, and Pt. This entails faradaic reactions at the electrode surface, continuously generating chemical products. To the best of our knowledge, no entirely ionic circuitry covering electrodeless ionic power source has been reported yet. Herein, we report a complete iontronic system comprised of ionic circuits and ionic power source for the first time with several noticeable features.

Reverse electrodialysis (RED) provides a direct route to power generation by converting the free energy of mixing two salt solutions in different concentration, e.g. seawater and river water which are inexhaustible resources at estuaries^24,25^. Assuming perfect permselectivity of an ion-exchange membrane (IEM), *ca*. 80 mV can result from preferential ion transport from seawater to river water (0.50 M and 0.017 M, respectively) when the IEM is present in between those solutions. Any arbitrary electric potential can be obtained by adjusting the number of IEMs and the salinity ratio of two salt solutions. Regarding the unique characteristic that the power generation process only involves ionic current, the RED system as an ionic power source would be suitable for the construction of a complete iontronic device.

**Figure 1:**
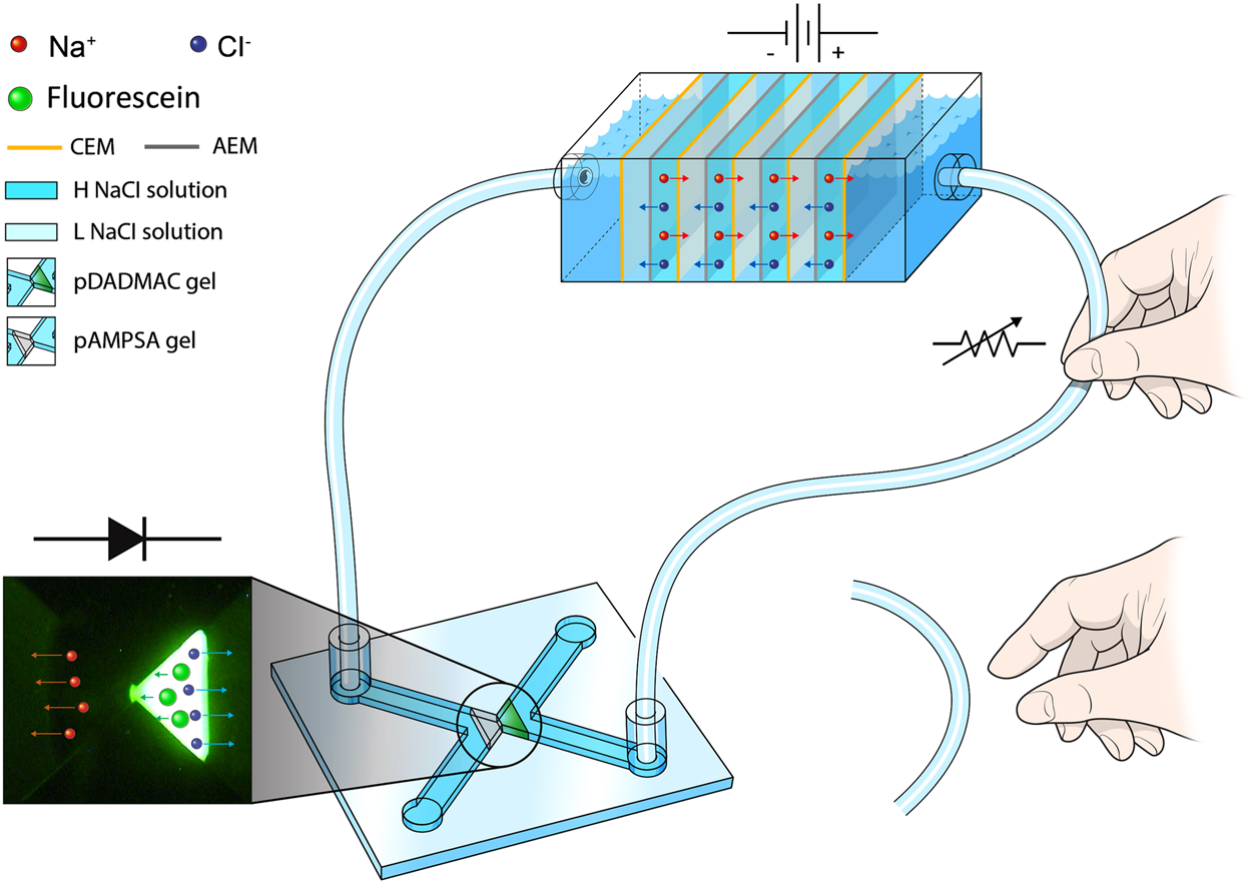
Illustrative schematic of ionic circuit powered by RED. Voltage generated from RED is applied to microfluidic polyelectrolyte diode directly via tubes filled with electrolyte. (CEM: Cation exchange membrane, AEM: Anion exchange membrane, H(L) NaCl solution: High(low) concentration NaCl solution).

In this work, we show the first example of ionic circuits driven by a miniaturized RED stack without using conventional electronic power sources such as battery or potentiostat. The schematic illustration of the proposed system is shown in Fig. 1. Ionic diode and logic gate circuit in a microfluidic chip are operated by salinity gradient power in the RED system. In addition, the voltage applied to the circuit can be turned on and off, and more precisely controlled by manipulating the flexible connection tube filled with electrolyte solution, which is reminiscent of a variable resistor.

## Results and Discussion

### Electrical characterization of RED

For construction of the system, a miniaturized RED stack was manufactured as an ionic power source^26^. The RED stack generated 2.2 V with 25 IEMs, which is 62.6% of theoretical value (3.51 V). This deviation was originated from imperfect permselectivity of the IEMs and additional conducting paths created by the solution feed channels in the RED system^27^. A higher RED voltage can be achieved through an optimization process of the stack configuration, e.g. solution paths for feeding, thickness of solution channels between IEMs, and salt concentration ratios. Nevertheless, in this work, we could readily obtain any RED voltage required for the ionic circuit operation.

### Ionic diode powered by RED

The microchip-based ionic diode employed in this study is similar to that introduced in our previous report^8,9^. A pair of positively charged poly(diallyldimethylammonium chloride) (pDADMAC) and negatively charged poly(2-acrylamido-2-methyl-1-propanesulfonic acid) (pAMPSA) is located at the center of the X-shaped microchip.

**Figure 2:**
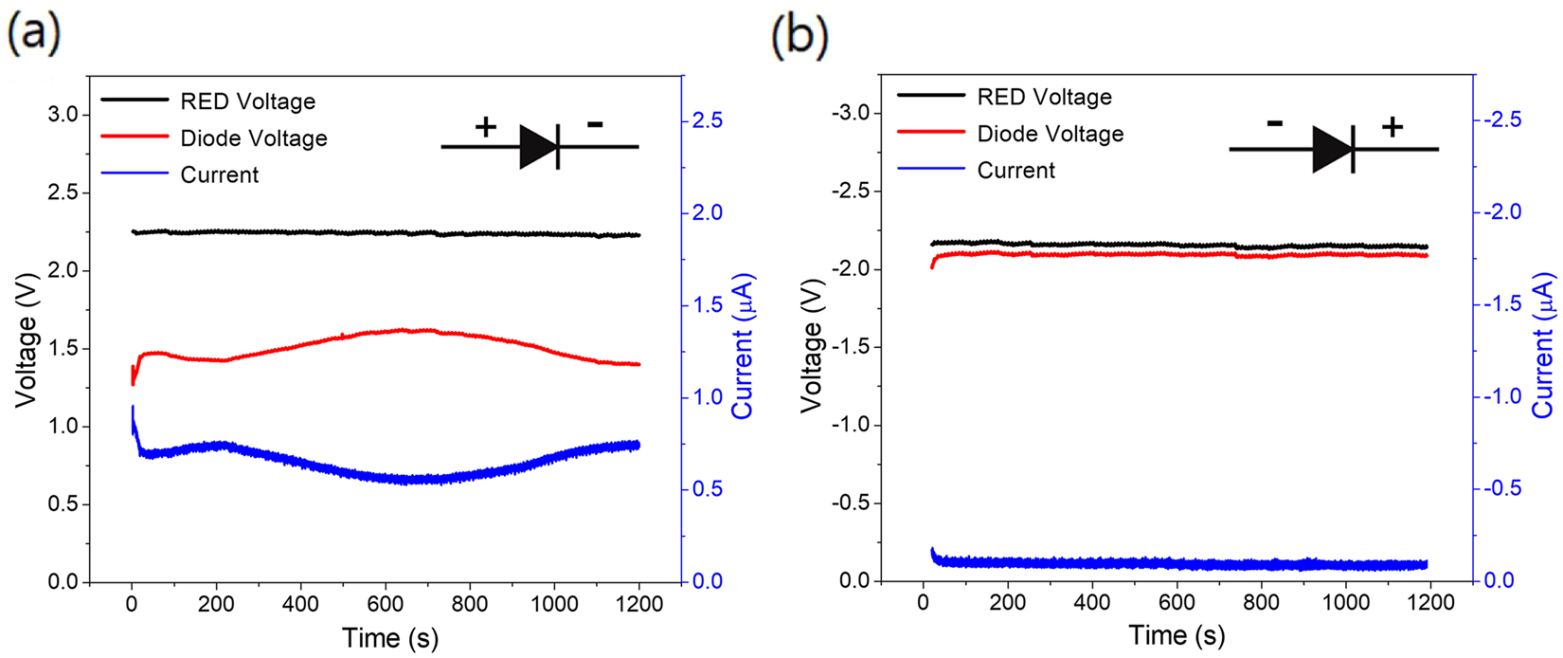
Time vs. voltage and ionic current plot of RED-powered ionic diode under (**a**) forward bias, and (**b**) reverse bias voltage. Note that the sign of voltage and current is inversed under reverse bias condition. (Black solid line: voltage generated from RED, Red solid line: voltage drop across the diode, Blue solid line: ionic current).

As shown in Fig. 1, we constructed an aqueous ionic diode circuit connected to RED stack through flexible, electrolyte-filled tubes involving no electronics. Both the reservoirs at the ends of the RED, the connection tubes and ionic circuits were filled with 10 mM NaCl solution containing 1 μM fluorescein to visualize the dynamic ion flow in the ionic diode in real-time. The voltage and current responses with time were measured for forward and reverse bias states of the ionic diode. (Fig. 2) The experimental scheme of voltage and current measurement is presented in Supplementary Fig. S1. Under forward bias, a voltage range of 1.4–1.6 V was imposed to the ionic diode with a current range of 0.6–0.7 μA while the RED voltage was maintained at 2.2 V (Fig. 2a). The remaining voltage (0.6–0.8 V) was consumed as an IR drop by the resistive parts such as connection tubes or reservoirs. Conversely, almost entire RED voltage was applied to the ionic diode under reverse bias while the ionic current was less than 0.1 μA (Fig. 2b). The results imply that the resistance of ionic circuit is significantly greater under reverse bias than under forward one due to the formation of an ion depletion region.

**Figure 3:**
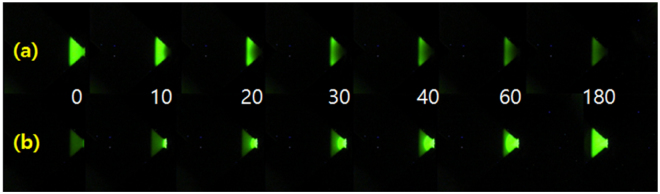
Temporal fluorescence images of n-type pDADMAC gel in ionic diode using anionic fluorescein under (**a**) forward bias potential, and (**b**) reverse bias potential. The white numbers in the middle of each pair of images represent the elapsed time in second after connection of RED to the circuit.

Figure 3 shows a series of temporal fluorescence images when either of the two biases is applied. Before applying forward bias potential, the n-type pDADMAC region exhibits a strong fluorescence because of the presence of anionic fluoresceins. When the diode is under forward bias, the fluoresceins in pDADMAC are gradually replaced by colorless Cl^−^ ions from the reservoir which contribute more efficiently to the forward ionic current with simultaneous reduction of fluorescence intensity. In contrast, the darkened pDADMAC gel starts to quickly recover its initial fluorescence intensity under reverse electrical bias as Cl^−^ ions inside are substituted with larger fluoresceins. We also conducted an additional experiment in order to examine the net effect of the reverse bias over no bias on the recovery of fluorescence intensity in the pDADMAC gel (Supplementary Fig. S2). This comparative experiment under confirms that the fluorescence intensity change is mainly affected by the reverse bias. Although the diode gradually brightens without any electrical bias, the speed of fluorescence recovery is significantly greater in the presence of reverse bias voltage. These results show that the aqueous ionic circuit can be successfully embodied with combination of the ionic power of RED.

### Voltage switching and regulation upon ionic circuit

**Figure 4:**
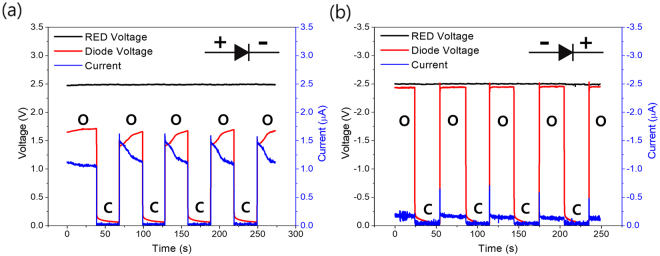
Time vs. voltage and ionic current plot for repetitive voltage switching upon ionic diode through mechanical pressure on flexible connection tube filled with electrolyte under (**a**) forward, and (**b**) reverse bias condition. The bold O and C on the graph indicate the open(on) and closed(off) tube states, Note that the sign of voltage and current is inversed under reverse. (Black solid line: voltage generated from RED, Red solid line: voltage drop across the diode, Blue solid line: ionic current).

The flexible connection tube filled with electrolyte solution plays dual roles: (1) connection of the RED to the ionic circuit, which is analogous to the conducting wire in electronics, and (2) a variable resistor when tightened or released at a specific point as described in Supplementary Fig. S3. Because the material resistance is inversely proportional to its cross section, the resistance of connection tube increases when it is constricted, and decreases when released. Thus, the voltage applied to the circuit is maximized when the tube is completely open whereas almost no voltage is applied when the tube is fully tightened. We measured voltage and current responses upon the pressure imposed to the tube for the forward- and reverse-biased ionic diode while the whole voltage from RED was almost kept constant at approximately 2.5 V (Fig. 4). Under forward bias, about 1.7 V was applied to the diode with a distinct current value (> 1.0 μA) when the tube was in the released state (Fig. 4a). However, when the tube was completely tightened, the applied voltage and current were drastically dropped to nearly zero. This phenomenon was reproducibly observed depending on the tube states. A similar result is shown in Fig. 4b for reverse-biased ionic diode. In this case, the majority of the RED voltage (approx. 2.45 V) was applied to the diode while the tube was open, since the resistance of the circuit (> 20 MΩ) is extremely higher than the other rest parts in the circuit (~ 400 kΩ) (e.g. reservoirs, electrolyte) by orders of magnitude. However, the applied voltage and ionic current dropped to nearly zero due to the infinitely increased tube resistance when the tube was fully constricted.

**Figure 5:**
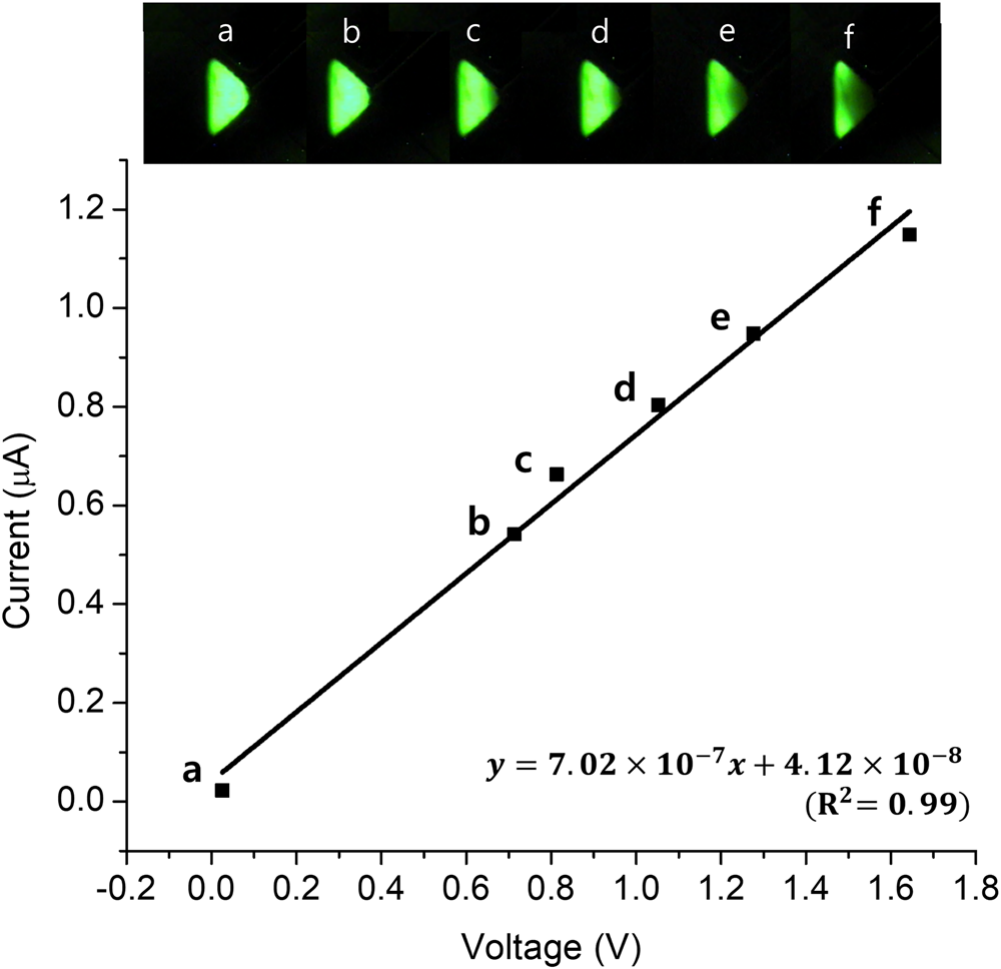
*I-V* plot for forward-biased ionic diode at steady state with fluorescence images of n-type pDADMAC gel for each state during the voltage adjustment. The voltage drop across the circuit (*V*) and ionic current (*I*) were measured 120 s after steady state had been formed.

An arbitrary potential beyond the only on and off states can be also applied to the ionic diode in a more controlled manner. For example, 6 different potentials including on and off states were applied to forward-biased ionic diode by precisely controlling the magnitude of the pressure onto the tube (Fig. 5). The steady-state ionic currents are directly proportional to the voltage drops across the circuit. The corresponding fluorescence images are in good agreement in that the fluorescence intensity gradually decreases with increasing applied voltage. These results demonstrate that we can tune the voltage applied to ionic circuits by using a variable-resistor-like tube filled with aqueous solution. As for reverse bias, only on and off states were allowed regardless of the magnitude of tube pressure. Neither voltage between the two states was stably maintained. As discussed in Supplementary Note 1, the tube resistance should surpass that of the circuit to adjust the potential applied to the diode even under reverse bias, which can reach several hundreds of MΩ according to the electrochemical impedance spectroscopy (EIS) analysis in the previous report^9^. More precise and delicate tools such as automatic micrometers and microvalves would enable finer control of the tube resistance to apply an arbitrary potential to any ionic circuit.

### Ionic OR gate driven by RED

**Figure 6:**
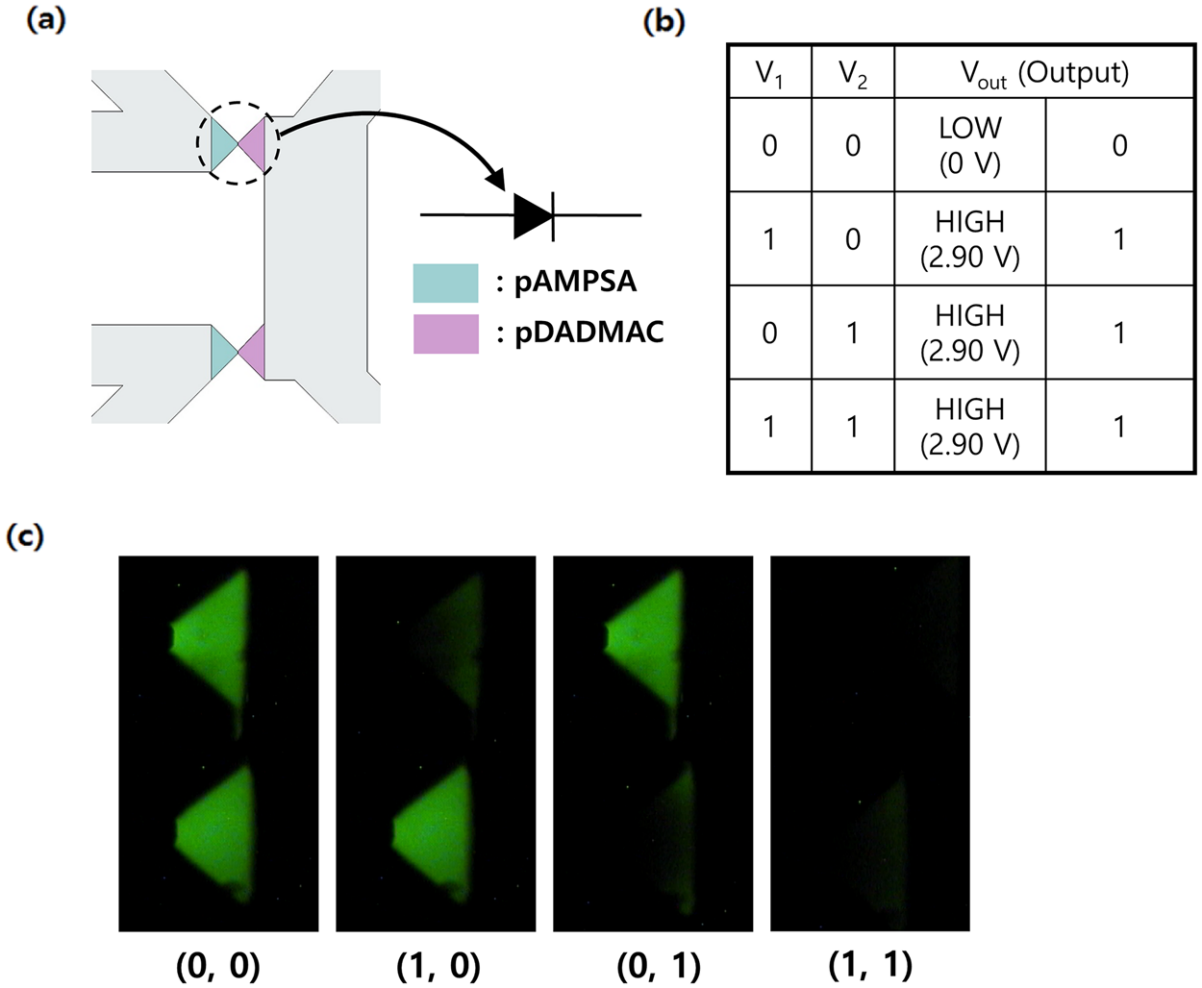
(**a**) Microchip pattern of ionic OR logic circuit, (**b**) Truth table of the voltages from RED and output signals from the circuit, (**c**) Fluorescence images for each state of the logic gate. Input voltage from RED was maintained at 3.1 V.

The concept of RED-powered iontronic system can be further expanded to a more sophisticated aqueous ionic circuit. Figure 6a shows an integrated microfluidic OR logic circuit composed of two ionic diodes in parallel (Supplementary Fig. S4). We obtained the truth table (Fig. 6b) of the circuit with fluorescence images using fluorescein (Fig. 6c), and output voltages for each state. The applied voltage becomes ‘0’ (off-state, low voltage) or ‘1’ (on-state, high voltage) as each tube is tightened or released at a specific point. The experimental results are in accordance with that of general digital OR gate in electronics in that the output voltage, which is defined as a voltage drop upon the circuit, reaches ‘1’ state when either of the two inputs is on ‘1’. When using anionic fluorescein for imaging the system, the fluorescence intensity in n-type pDADMAC phase markedly decreases in forward-biased (‘1’ state) state. Consequently, RED in aqueous media can supply power to a more elaborate information processor in microfluidic chip.

## Conclusions

In conclusion, we have demonstrated that ionic circuits can be successfully driven with combination of RED in a fully ionic and electrodeless manner with several noticeable features such as voltage switching and control. This unique characteristic is particularly important because one of the prospective potentials of iontronics is to devise ionic information processors and intrinsically biocompatible interface with *in vivo* system such as neural network. With regard to neural engineering, a full ionic system can make sensing and stimulating perfectly free from faradaic reaction at electrode surface. Being a proof-of-concept without much sophistication yet, the proposed system would suggest various applications where an aqueous, ionic, and non-metallic power source are required such as a bio-inspired information processor. Additionally, there are many possibilities to ameliorate the system in future studies by optimizing device size, miniaturizing via integration of RED part into a single microchip, or inventing a method for finer control of the voltage imposed to the circuit.

## Materials and Methods

### Materials

All chemicals were used without further purification. Diallyldimethylammonium chloride (DADMAC), 2-acrylamido-2-methyl-1-propanesulfonic acid (AMPSA), sodium chloride, potassium chloride, 2-hydroxy-4ʹ-(2-hydroxyethoxy)−2-methylpropiophenone, fluorescein sodium salt, 3-(trimethoxysilyl)propyl methacrylate (TMSMA), methanol, and Ag wire (0.5 mm thick, 99.9%) were purchased from Aldrich. Selemion CMV and AMV were purchased from Asashi Glass Co., Ltd.

### Preparation of RED stack

We manufactured a customized RED stack by mostly following the methods of M. C. Hatzell and B. E. Logan^26^. Briefly, silicon gaskets (~1.3 mm in thickness) were cut into a rectangular shape (2 × 1 cm^2^) to provide flow path between IEMs that had a cross section area of 2 cm^2^. The silicon gaskets and IEMs as prepared were stacked up in a repetitive alternation (i.e. CEM, gasket, AEM and gasket, respectively) and then, two solution chambers for external connection to ionic circuit were attached to the lateral ends of the stack comprising gaskets and IEMs. Two salt solutions (0.01 and 4.0 M NaCl) were introduced into the RED system with a flow rate of 0.71 mL/min. Typically, the RED stack generated 2.2 V under those conditions.

### Microchip fabrication

The microchips were fabricated by following the previously reported procedure^8^. Corning 2947 precleaned slide glasses (75 mm × 25 mm, 1 mm thick, Corning, USA) were used as substrates. The slide glass was cleaned in a piranha solution (H_2_SO_4_:H_2_O_2_ = 3:1, J.T. Baker, USA) for 45 min and then rinsed with deionized (DI) water (NANOpure Diamond, Barnstead, USA) several times. After removing the moisture on the surface with an air blower, the cleaned slide glass was dehydrated on a hot plate at 200 °C for 5 min and then cooled to room temperature. The slide was then spin-coated (YS-100MD, Won Corp., Korea) with hexamethyldisilazane (HMDS; Clariant, Switzerland) at 7000 rpm for 30 s. It was then coated with a photoresist (PR; AZ4620, Clariant, Switzerland) at 7000 rpm for 30 s. After soft baking the PR on a hot plate at 100 °C for 90 s, the slide glass was cooled to room temperature and aligned under a pattern mask. The PR on the slide was exposed to UV light (365 nm) with an intensity of 21 mW cm^−2^ for 13 s (MDE-4000, Midas, Korea) at AZ 400 K developer (Clariant, Switzerland) for 120 s. The slide glass was then washed with DI water, and the PR was hard-baked on a hot plate at 200 °C for 15 min. Adhesion tape was attached to the back side of the slide glass in order to protect it from the etching solution. The slide glass was etched with a 6:1 buffered oxide etch solution (J. T. Baker, USA) for 45 min at 25 °C with stirring. The etched glass was then drilled at the positions for the reservoirs with a 2 mm-diameter diamond drill at 18000 rpm and cleaned in a piranha solution for the same duration. The pair of slide glasses were permanently attached to each other by thermal bonding. DI water between the glasses prevented the formation of air bubbles during the bonding process. The glasses were heated at 600 °C in a furnace (CRF-M15, Ceber, Korea) for 6 h at which time they were slowly cooled in the furnace to room temperature over 10 h.

### Fabrication of polyelectrolyte ionic diode in microchip

Diallyldimethylammonium chloride (DADMAC) and 2-acrylamido-2-methyl-1-propanesulfonic acid (AMPSA) were used as the monomers to create positively and negatively charged polyelectrolytes. Before gelation, the microchannel was coated with 3-(trimethoxysilyl)propyl methacrylate (TMSMA, 0.5%) in a methanol solution containing acetic acid (0.5%) for 1.5 h. TMSMA acted as the linker between the polyelectrolyte and the slide glass surface. The microchannel was then cleaned with methanol. The microchannel was aligned under a mask and subsequently exposed to UV light (365 nm) with an intensity of 21 mW cm^−2^ for 3.5 s (MDE-4000, Midas, Korea). After photopolymerization, the microchannel was cleaned with KCl (1 M) to remove the remaining DADMAC monomers, photoinitiator, and cross-linker (2%). The microchip with the pDADMAC gel plug was then filled with a AMPSA (5 M) solution containing a photoinitiator (2%) and a cross-linker (2%). After fine alignment under the mask, the chip was exposed to UV light for 9 s to produce a sharp polyelectrolyte junction. The microchannel was then washed with KCl (1 M). Finally, the polyelectrolyte junction comprising pDADMAC and pAMPSA on the microchip was stored in aqueous NaCl (10 mM).

### Connection of RED to the ionic circuit

Cloning cylinder (H 6 mm × 8 mm, Aldrich) was attached through epoxy-resin bonding at each position of reservoirs of the fabricated microchip. An end of the plastic tube (AJK00004, Tygon chemical resistant tubing, Saint-Gobain) filled with electrolyte was then inserted to the bonded cylinders, and the other end was located inside the solution reservoir of RED. The electrolyte employed for connecting and constructing ionic circuit is 10 mM NaCl solution. The whole system was constructed without any air bubble inside the connection tubes.

### Voltage and current measurements

The whole system does not include any metallic electrode, nor an electronic instrumentation such as potentiostat for monitoring voltage and current signals in the ionic circuit. Instead, a multichannel basic data acquisition device (USB-6003, National Instruments, USA) of 16-bit resolution was utilized for voltage and current recording. As depicted in Supplementary Fig. S1, each analog input channel was allocated for measuring a electrical potential difference between two arbitrary points. For example, the voltage generated from RED was measured by dipping two Ag/AgCl electrodes in both the reservoirs at each end of the RED. Similarly, the voltage drop across the ionic circuit was measured with the same method at analog channel 1. Meanwhile, the ionic current flowing through the circuit was indirectly calculated from the potential drop across the electrolyte-filled connection tube whose resistance had been measured in advance. This follows simple Ohm’s law (Equation (1)),

1 $${\bf{I}}({\bf{current}})=\frac{{{\boldsymbol{V}}}_{{\boldsymbol{diff}}}}{{{\boldsymbol{R}}}_{{\boldsymbol{tube}}}}$$

where *V*_diff_ and *R*_tube_ represent the voltage difference between two ends of a tube and its resistance. This current measuring method is possible because the resistance of an electrolyte-filled tube reaches several hundreds of kΩ, whereas the conducting wire in electronic circuits is *de facto* negligible in terms of resistance. These voltage data were simultaneously acquired with sampling rate of 100 samples per second.

### Optical measurement

For light-emission experiment, the fluorescence from the n-type polyelectrolyte gel, pDADMAC, was observed while the ionic circuit was driven by RED. For this purpose, aqueous NaCl solution (10 mM) containing 1 μM of fluorescein was used as electrolyte in the ionic circuit. The fluorescence intensity was measured by a fluorescence microscope (TE2000U, Nikon, Japan).

## Electronic supplementary material

Supplementary Information
