# Supplementary material for: Ionic Circuits Powered by Reverse Electrodialysis for an Ultimate Iontronic System
Source: Sci Rep. 2017 Oct 25;7:14068. doi: 10.1038/s41598-017-14390-0 (PMC5656583; doi:10.1038/s41598-017-14390-0)
Supplement: Supplementary file 1 — Supplementary Information [file 41598_2017_14390_MOESM1_ESM.pdf]

Supporting information

Ionic Circuits Powered by Reverse Electrodialysis for an Ultimate Iontronic System

Seok Hee Han[1§], Seung-Ryong Kwon[1§], Seol Baek[1] and Taek-Dong Chung[1,2]*

[1]*Department of Chemistry, Seoul National University, Seoul 08826, Korea*

[2]*Advanced Institutes of Convergence Technology, Suwon-Si, Gyeonggi-do 16229, Korea*

[§]Both authors contributed equally to this work.

* Corresponding author: Prof. Taek-Dong Chung, tdchung@snu.ac.kr

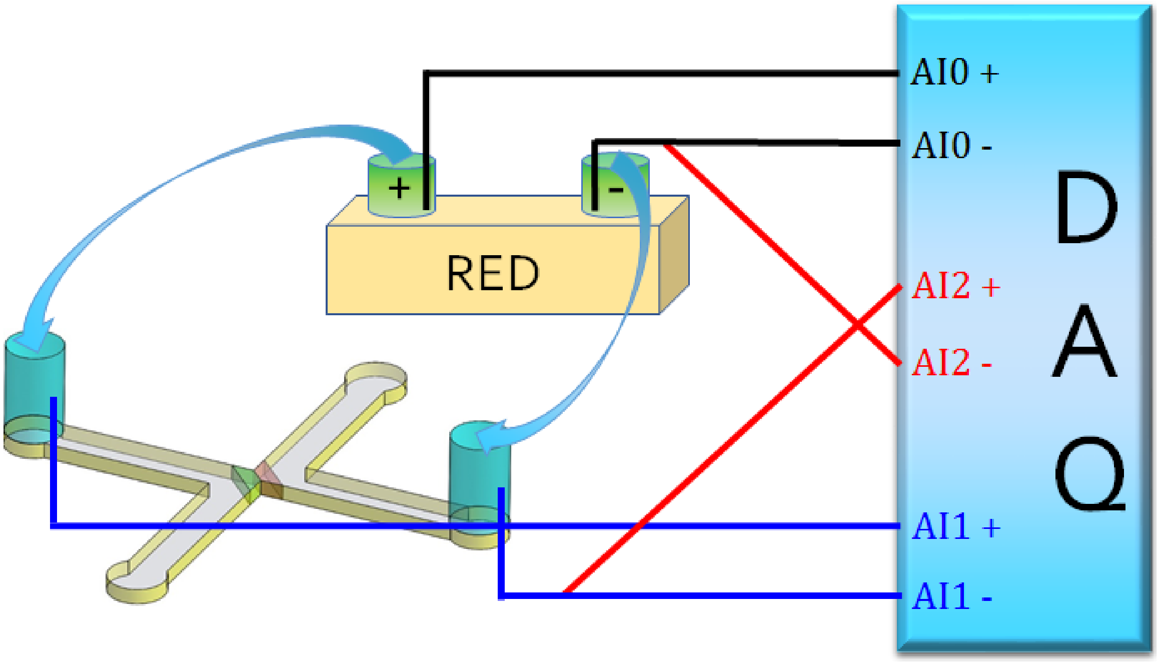

Figure S1. Experimental scheme of voltage and current measurements using data acquisition (DAQ) device. AIs indicate analog input channels of the device, each pair of which is composed of + and – terminals. The electrical potential difference between these two points is recorded constantly during the experiment.

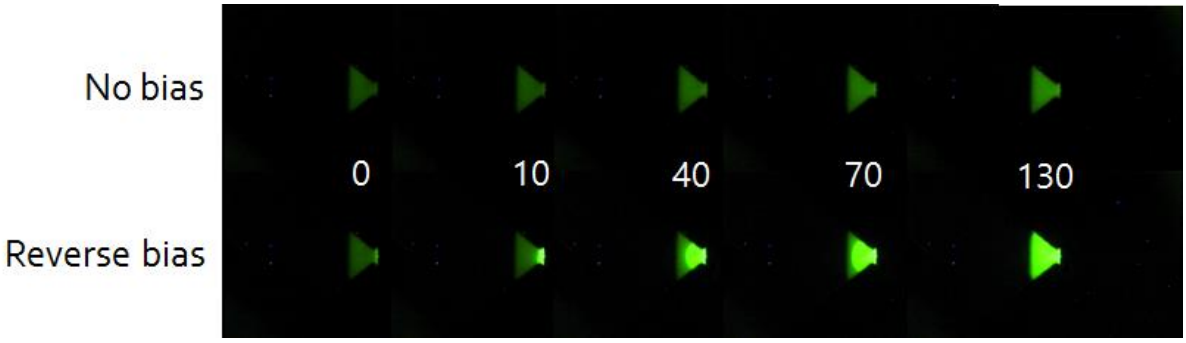

Figure S2. Comparison between temporal fluorescence images of pDADMAC gel under no external bias and reverse bias potential. The white numbers in the middle of each pair of images indicate the elapsed time in second from the initial state.

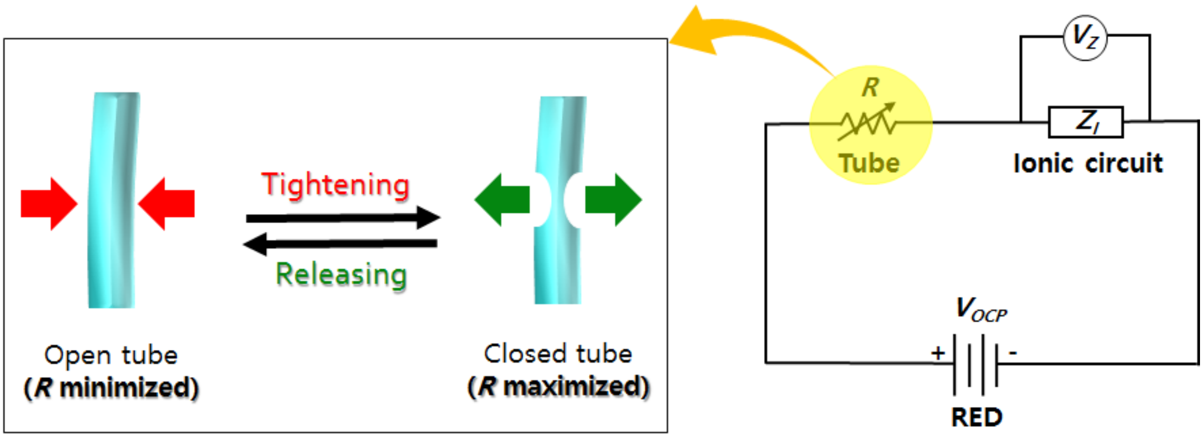

Figure S3. Schematic diagram of flexible plastic connection tube tightened or released by external pressure with a simplified equivalent circuit of the system. (R: Resistance at the specific point where the tube is tightened or released, V_Z: Voltage drop across the ionic circuit, Z_I: Total impedance (resistance) of the ionic circuit, V_{OCP}: The entire voltage generated from RED)

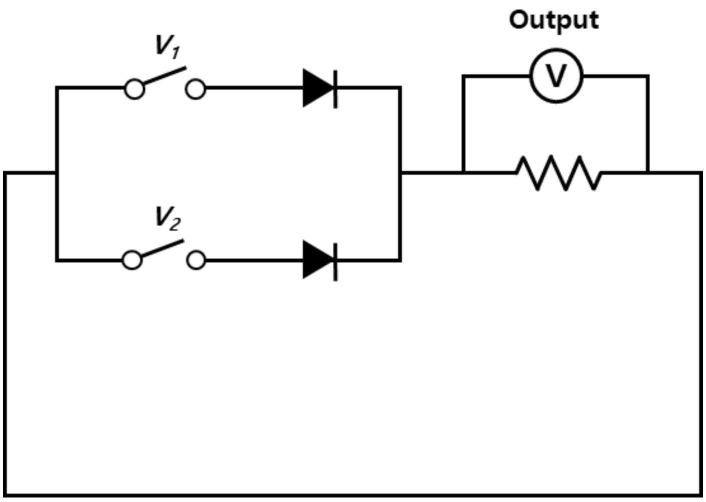

Figure S4. Equivalent circuit of ionic OR logic gate. V_1 and V_2 represent the input voltages originated from RED which are switched on and off by manipulating connection tubes. The output voltage is defined as a voltage drop across the resistive component of the circuit following each ionic diode.

Note 1. Voltage controllability upon ionic circuits using flexible connection tube

Theoretically, in turn, the tube can also be utilized as a variable resistor if it is partially constricted in a precise and delicate manner (see Supplementary Fig. S3). However, we observed that the voltage applied to the reverse-biased ionic diode was not stably maintained for more than a few seconds. Whichever the desired voltage is, it quickly returned to its initial value ("on" or "off" state). This phenomenon in all-or-nothing fashion under reverse bias condition can be further examined by a simple voltage-divider equation (Equation (1)),

$$\boldsymbol{V_Z(R) = V_{OCP}\frac{Z_I}{R+Z_I}} \qquad (1)$$

where V_Z represents the voltage drop across the ionic circuit, V_{OCP} the voltage generated from RED, Z_I the circuit resistance, and R the resistance of other circuit components except the main circuit (e.g. ion diode). As V_{OCP} and Z_I are constant values for a certain circumstance, V_Z is a function of the variable resistance R, which is controlled by the tube constriction method. When $Z_I \gg R$, V_Z is effectively equal to V_{OCP} as in the reverse biased ionic diode where the resistance of diode junction can surpass several hundreds of MΩ due to an ion depletion region. Even though V_Z may decrease if R becomes sufficiently large to compete with Z_I as the tube is tightened enough, the problem is that the tube is already effectively closed for resistance to reach that of ion depletion region in reverse-biased ionic diode.
